# Multiplex antibiotic susceptibility testing of urinary tract infections using an electrochemical lab-on-a-chip

**DOI:** 10.1007/s10544-024-00719-w

**Published:** 2024-08-09

**Authors:** Benjamin Crane, Alex Iles, Craig E. Banks, Mamun Rashid, Patricia E. Linton, Kirsty J. Shaw

**Affiliations:** 1 Department of Natural Sciences, Manchester Metropolitan University, Manchester, UK; 2 Department of Materials & Environmental Chemistry, University of Stockholm, Stockholm, Sweden; 3 Previously at Faculty of Science & Engineering, University of Hull, Hull, UK

**Keywords:** Antibiotic susceptibility testing, Bacteria, Hydrogels, Infection, Screen-printed electrodes

## Abstract

Urinary tract infections (UTIs) represent the most prevalent type of outpatient infection, with significant adverse health and economic burdens. Current culture-based antibiotic susceptibility testing can take up to 72 h resulting in ineffective prescription of broad-spectrum antibiotics, poor clinical outcomes and development of further antibiotic resistance. We report an electrochemical lab-on-a-chip (LOC) for testing samples against seven clinically-relevant antibiotics. The LOC contained eight chambers, each housing an antibiotic-loaded hydrogel (cephalexin, ceftriaxone, colistin, gentamicin, piperacillin, trimethoprim, vancomycin) or antibiotic-free control, alongside a resazurin bulk-modified screen-printed electrode for electrochemical detection of metabolically active bacteria using differential pulse voltammetry. Antibiotic susceptibility in simulated UTI samples or donated human urine with either *Escherichia coli* or *Klebsiella pneumoniae* could be established within 85 min. Incorporating electrochemical detection onto a LOC provides an inexpensive, simple method for the sensitive determination of antibiotic susceptibility that is significantly faster than using a culture-based approach.

**Supplementary Information:**

The online version contains supplementary material available at 10.1007/s10544-024-00719-w.

## Introduction

Urinary tract infections (UTIs) represent the most prevalent type of outpatient infection, affecting up to 60% of women in their lifetime, and have a significant burden of morbidity and mortality in the elderly (Medina and Castillo-Pino 2019). There are also significant effects on quality-of-life measures in women suffering from recurrent UTIs and economic impacts with a cost of ~$1.6 billion annually in the US alone. UTIs are precipitated by the colonization of the urethra followed by migration of the bacteria up the urinary tract to the bladder (Flores-Mireles et al. 2015). Health complications can arise from complicated UTIs as further bacterial colonisation from the bladder to the simple epithelium in the kidneys occurs. From here, bacteria can migrate across the tubular epithelial barrier and enter the blood stream causing bacteraemia (Alqarni et al. 2018). Bacteraemia results in urosepsis which accounts for 25% of sepsis cases in adults with an associated mortality rate of 25–60% (Peach et al. 2016).

UTIs are often diagnosed using a dipstick test followed by culture-based antibiotic susceptibility testing (AST), with broad spectrum antibiotics commonly prescribed before AST results are obtained as this can take up to 72 h (Pfaller and Jones 2006). This can result in ineffective antibiotic prescription which can lead to poor clinical outcomes and development of further antibiotic resistance. Therefore, the development of more rapid AST methods is important to improve initial prescription of effective antibiotics to improve treatment (Issakhanian and Behzadi 2019). For example, the move from phenotypic to genotypic testing using molecular methods such as the polymerase chain reaction (PCR) can identify resistance genes in the pathogen more rapidly, within a few hours (Eigner et al. 2005; Thomson et al. 2007), but there are challenges due to the potential for multiple resistance genes to be present (van Hoek et al. 2011), and the fact that the genes may not confer phenotypic resistance (Davies and Davies 2010).

A review of rapid diagnostics for antimicrobial resistance presented a range of genotypic and phenotypic techniques and their associated challenges at the point-of-care level which commonly include overall cost and regulatory bottlenecks (Shanmugakani et al. 2020). More recently, lab-on-a-chip (LOC) devices have been explored as a potential method for conducting AST (Postek et al. 2022). LOC technology uses microfluidics to enable the miniaturization and integration of bioanalytical techniques, offering advantages in terms of increased portability, reduced cost, and rapid analysis times. A variety of different approaches for analysing suspected UTI samples have been investigated and can be classified according to the methods used for reporting bacterial response, such as optical density (OD) measurement (Wityk et al. 2023), fluorescent detection (Wu et al. 2022, 2023), advanced spectroscopic techniques (Ramzan et al. 2024), optical assays (Xu et al. 2023), and electrochemical sensing (Huynh et al. 2023).

Electrochemical methods used one of three approaches; amperometry, the measurement of the current, at a fixed potential, generated at the electrode to detect electrically active metabolites released by bacteria (Kotanen et al. 2012); impedance, to monitor changes in the resistivity or motility of any bacteria present (Safavieh et al. 2017); or differential pulse voltammetry, used in the detection of redox markers to identify metabolically active bacteria (Besant et al. 2015). Of these three methods, the latter represents a label free method which offers advantages in terms of shorter analysis times, cost-effectiveness and sensitivity (Luppa et al. 2001; Hai et al. 2020), and therefore has the greatest potential for rapid AST in urine samples.

Reagent storage is another very important aspect to consider in the generation of sample in-answer out systems that can be used outside of a conventional laboratory setting. Hydrogels, cross-linked hydrophilic polymers suspended in water, have a documented history of use as a delivery vector for drugs in a number of applications, including for use with antibiotics (Ahmadian et al. 2022). Poly(vinyl alcohol) (PVA) based hydrogels have good mechanical properties, are stable at room temperature, have a high water content when hydrated, and have no significant negative effect on the growth of bacteria (Ricciardi et al. 2005) so are ideal for antibiotic susceptibility testing.

Here we present a cost-effective microfluidic device, incorporating screen-printed electrodes (SPEs), capable of multiplex AST of seven clinically relevant antibiotics in less than 90 min. An evaluation of the SPEs and hydrogels for antibiotic storage and release is presented, alongside performance of the LOC devices with simulated UTI samples from both artificial and human urine samples.

## Experimental

### Biological samples

*Escherichia coli (E. coli)* ATCC 25922 (vancomycin resistant), *Klebsiella pneumoniae (K. pneumoniae)* ATCC 700603 (vancomycin resistant) or *E. coli* NCTC 13351 (cephalexin, ceftriaxone and vancomycin resistant) were tested as model and clinically relevant Gram-negative bacteria that are commonly present in UTIs (Terlizzi et al. 2017). Overnight cultures were prepared in single strength nutrient broth (SM0002 (Thermo Scientific, UK) and incubated at 37 °C. The optical density was then adjusted to 0.7 at 600 nm using a spectrophotometer. Cultures were then pelleted and resuspended in either artificial urine (made of equal volumes of surine artificial urine (Sigma-Aldrich, UK) and double strength nutrient broth, adjusted to pH 6 and sterilised via autoclave) or donated urine samples at a concentration of 1.75 × 10^8^ colony forming units (CFU)/mL. Urine samples were donated by healthy volunteers. The pH of the urine was recorded, the samples were sterilised via autoclave, and then mixed at a 1:1 ratio with double strength nutrient broth.

### Microbiology analysis

CFU/mL values were determined using standard microbiology techniques. Serial dilutions were performed with phosphate buffer saline (PBS) (Sigma-Aldrich, UK) and 100 µL samples were spread on agar plates, and incubated at 37 °C for 24 h, before being counted. Minimum inhibitory concentration (MIC) values for each of the antibiotics tested were confirmed against each of the three bacterial strains using a broth microdilution assay performed in triplicate using flat bottomed 96 well plates (Sarstedt, Germany). All antibiotics were solubilised in sterile deionised water, and filter sterilised using 0.2 μm syringe filter, at a concentration of 1000 µg/mL. The antibiotics were then serially diluted two-fold to a final concentration of 1.95 µg/mL in Mueller Hinton broth (Sigma-Aldrich, UK). Bacteria were added to the well at a concentration of 2 × 10^6^ CFU/mL and the optical density read on a Multiscan GO 96 well plate reader after 24 h incubation at 600 nm. The MIC value was taken as the lowest concentration which significantly inhibited observed bacterial growth.

### LOC devices

**Fig. 1:**
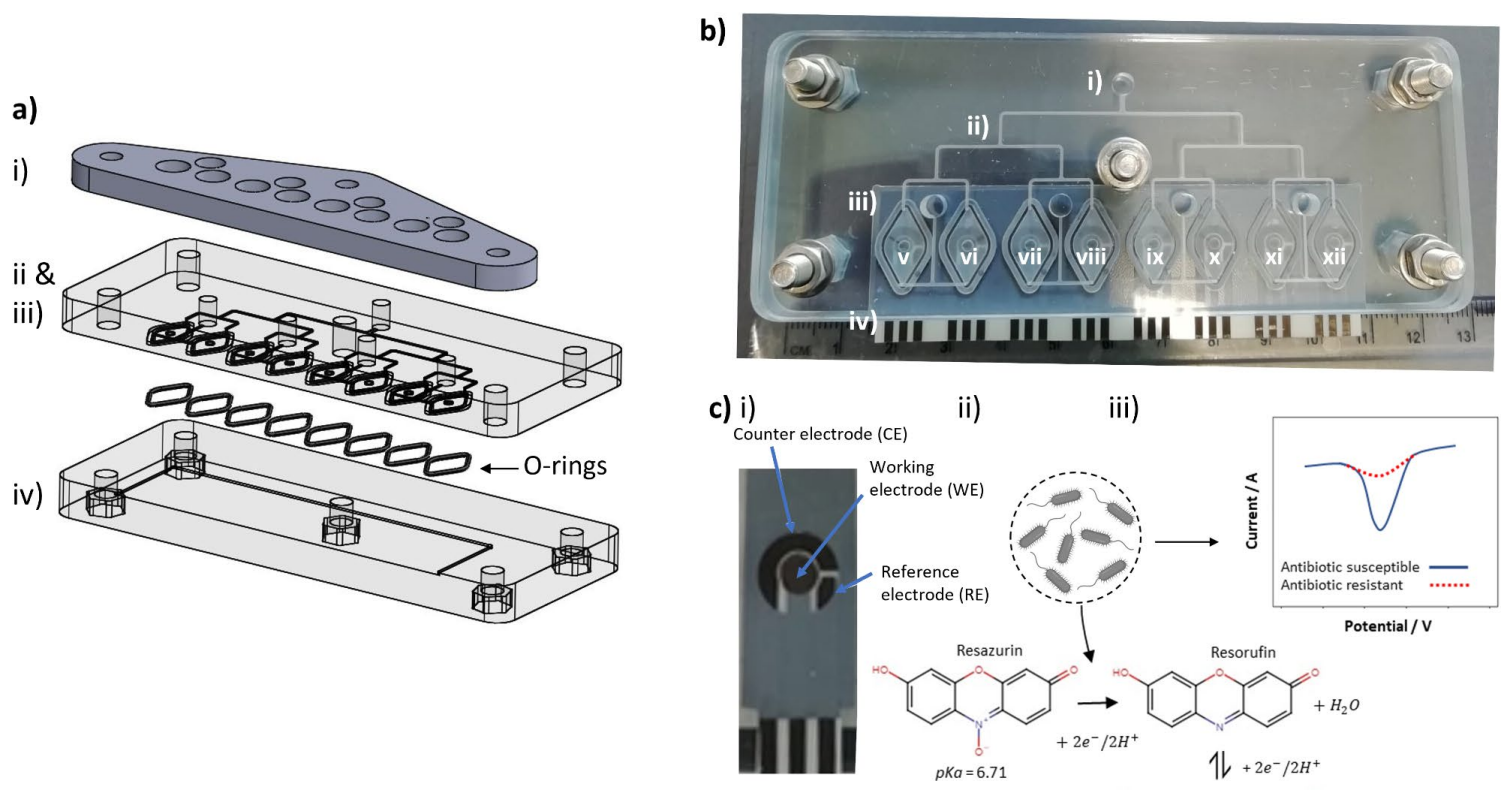
**(A)** CAD drawing of the overall LOC design, showing (i) aluminium plate, (ii) upper and middle polycarbonate layers containing inlet/outlet ports, microfluidic channels and chambers; and (iii) bottom polycarbonate layer with recess for the integration of SPEs; **(B)** Photograph of the completed device: (i) urine sample is loaded, (ii) microfluidic channels split the sample into eight chambers (iii) containing incorporating SPEs (iv). Chamber v) acts as the control and containing an antibiotic-free hydrogel, while the subsequent chambers contain different antibiotic-loaded hydrogels: ceftriaxone (vi), cephalexin (vii), colistin (viii), gentamicin (ix), piperacillin (x), trimethoprim (xi) and vancomycin (xii) (N.B. aluminium clamping layer excluded for clarity); **(C)** (i) Photograph showing a close up of an individual SPE, (ii) schematic of the principles of detection whereby resazurin is reduced to resorufin at the working electrode surface by metabolically active bacteria; and (iii) representation of how this is detected electrochemically

LOC devices were designed in AutoCAD and SolidWorks, and then manufactured in polycarbonate and aluminium using precision computerised numerical control (CNC) machining on a Datron M7 milling machine to produce the design shown in Fig. 1a. The devices consisted of four layers: (i) A 6 mm thick machined aluminium plate to spread the clamping force applied by the bolts to ensure sealing of all 8 chambers; (ii) An upper polycarbonate layer with holes milled for the inlet and outlet ports, and the microfluidic channels (width 500 μm, depth 300 μm); (iii) A middle 2 mm thick polycarbonate layer with through holes to eight rhomboid-shaped chambers (width 7 mm, length 13 mm, height 300 μm, total volume 16.5 µL). Each chamber contained a circular recess for the hydrogel and a recess for the O-rings (i.d. 10.5 mm, cross-section 1 mm (Polymax, UK)) to seal the chamber against the SPE; (iv) A 9 mm thick bottom polycarbonate layer with recess for the integration of SPEs, as well as hexagonal recesses on the underside for the M5, A2 stainless steel bolts used to clamp the device together. The middle and upper polycarbonate layers were aligned and fusion bonded using a DrCollin P200 hot press. Prior to first use, and in between samples, the LOC devices were cleaned with 70% (v/v) ethanol (Sigma-Aldrich, UK) for 10 min and air dried. A row of 8 SPEs was then added, the hydrogel discs loaded and the full LOC assembled. The LOC devices were filled using a model MD-1001 syringe pump (Bioanalytical Systems, USA), to which a 5 mL syringe (Sarstedt, Germany) was connected to the device via silicone tubing (o.d. 1 mm (Gardiflex, UK)), and liquid pumped at a rate of 10 µL/min.

Hydrogels were fabricated using a polyvinyl alcohol (PVA) Mw 89,000 to 99,000 (99+% hydrolysed, product number 341584, Sigma-Aldrich, Gillingham, UK) precursor which was made by adding 1 g of PVA to 10 mL of deionised water to produce a weight to volume ratio of 10%. The precursor was then autoclaved for one hour (Imtiaz et al. 2019) at 121 °C to sterilise the PVA and dissolve it completely. The precursor was then stirred continuously for 30 min at 50 °C and then cooled, at which point the relevant antibiotic was added to produce final concentrations of 0.8 mg/mL ceftriaxone, 2.6 mg/mL cephalexin, colistin 2.2 mg/mL, 5.6 mg/mL gentamicin sulphate, 4.2 mg/mL piperacillin sodium salt, 10.5 mg /mL trimethoprim and 45 mg/mL vancomycin, respectively. These concentrations were based on previously determined MIC data and a release factor of 40% over 20 min. All antibiotics were obtained from Sigma-Aldrich, UK. Due to the poor water solubility of trimethoprim, it was first solubilised in 0.1% dimethyl sulfoxide (DMSO) before being added to the hydrogel precursor. A control hydrogel was also produced which contained no antibiotic, both with and without DMSO present. The hydrogel precursors were then sonicated before being cast into a 0.3 mm thick layer. The hydrogels were then formed using eight freeze thaw cycles. The first cycle was made up of a -20 °C freeze cycle overnight followed by thawing at room temperature for 30 min. The remaining 7 cycles consisted of one hour of freezing at -20 °C and then thawing for 30 min at room temperature (Cascone et al. 2004). After the last freeze thaw cycle, the hydrogels were cut into 2 mm diameter working sections using a biopsy punch and added to the recesses in the relevant chambers on the LOC device (Fig. 1biii).

Resazurin (7-Hydroxy-3H-phenoxazin-3-one-10-oxide sodium salt) bulk-modified screen-printed macroelectrodes (R-SPEs) were produced using the methodology previously described (Crane et al. 2021). The inclusion of resazurin enabled identification of metabolically active bacteria due to their ability to reduce it to form resorufin, which can be detected using differential pulse voltammetry (DPV) (Fig. 1ci). The R-SPEs comprises working, counter and reference electrodes made from carbon ink, where the working electrode has a diameter of 3.1 mm. Note that these were made to have shorter connection lengths to those previously described which gives rises to improved heterogeneous electrode kinetics via resistance changes (Whittingham et al. 2021). The R-SPEs are added as a sheet of eight, enabling each reaction chamber to be monitored.

### Electrochemical measurement

Samples were pre-incubated for 70-minutes at 37 ºC and then applied to the LOC. After 15 min in the LOC, DPV was used to record resazurin reduction peak height for each of the reaction chambers. SPEs were interfaced with a EmStat3 potentiostat controlled by PSTrace 5.5 software using an EmStat three pin screen printed electrode connector (PalmSens, The Netherlands, Randhoeve). Cyclic voltammetry (CV) was conducted using an E– step of 0.005 V. The following scanning rates were used; 0.005, 0.01, 0.015, 0.025, 0.050, 0.075, 0.100, 0.150 and 0.200 V/s. Five replicates were performed per applied scanning rate. DPV experiments utilised an E-step of 0.001 V, a T-pulse of 0.05 s, an E– pulse of 0.05 V and a scan rate of 0.01 V/s. The resazurin I_pc_ (cathodic/reduction peak current) at each time point was calculated after the completion of the experiment. The height of the resazurin reduction peaks of the antibiotic treated artificial urine where statistically compared to those of the artificial urine with no antibiotic. A higher resazurin reduction peak height for the antibiotic treated artificial urine is associated with higher inhibition of bacterial growth. Five replicates were carried out per incubation time point. DPV measurements were taken using two matching potentiostats used simultaneously, recording in pairs (C1 and C5; C2 and C6; C3 and C7; and C4 and C8) taking a total of ~ 3 min.

### Statistical analysis

Statistical analysis was conducted using GraphPad Prism (Version 9.4.1) using 95% confidence limits. Normality of a dataset was assessed using a Shapiro-Wilk test. When comparing two data sets normally distributed data was analysed with a T-test. A Wilcoxon rank test was applied if normality was not determined. If more than two datasets required comparison, an ANOVA was used for normally distributed data with a post hoc Tukey test to establish which data was and was not significant. For non-normal data sets a Kruskal Wallis test was used with a follow up pairwise Wilcoxon test to determine which data was or was not significant. Regression was used to establish if there was a cause-and-effect relationship between two variables.

## Results

### Antibiotic hydrogels

PVA hydrogels were evaluated to determine their efficacy of antibiotic release, and to ensure the native hydrogels or presence of DMSO did not cause any inhibition of bacterial growth. Significant inhibition of bacterial growth was determined to occur within 15 to 20 min from antibiotic containing hydrogel pellets compared to antibiotic free controls (Fig. SI_1). The inhibitory effect of the antibiotics in the hydrogels was not instantaneous, with the antibiotic loaded hydrogels showing a steady release over time. Therefore, a time frame of 15 to 20 min for the release of the antibiotic was established before readings were taken. No inhibition of bacterial growth was caused by the presence of the antibiotic free hydrogels or DMSO. Vancomycin was also included as an example antibiotic which the bacterial strains are not susceptible to, and this showed that the electrochemical AST method could differentiate between effective and ineffective antibiotics.

**Fig. 2:**
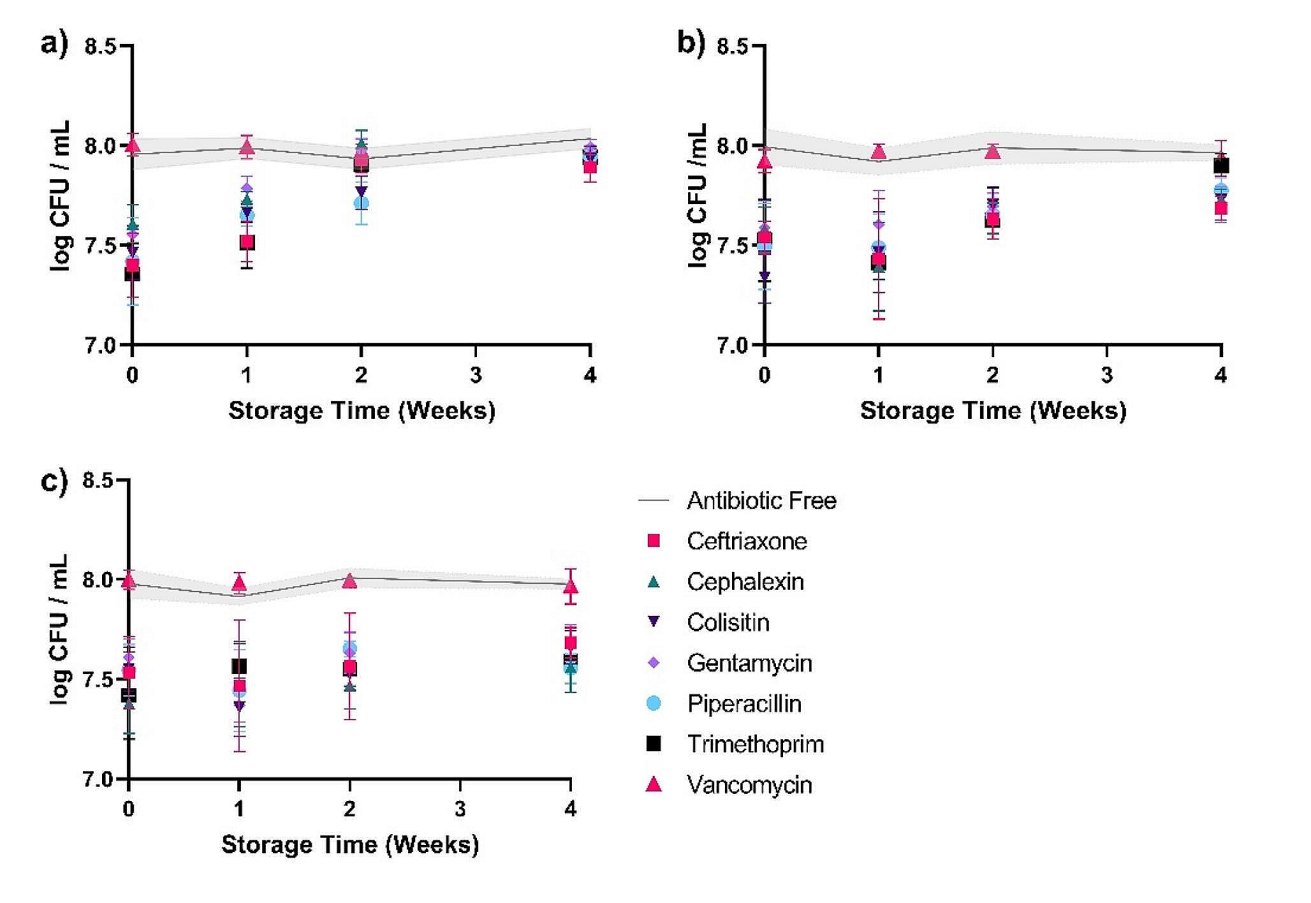
Effectiveness of each of the antibiotic hydrogels against *E. coli* ATCC 25922 after storage for different time periods at room temperature **(A)**, in the fridge **(B)** and in the freezer **(C)** compared to antibiotic free controls (*n* = 4)

The stability of the hydrogels was also determined when kept at room temperature, fridge and freezer conditions for up to 4 weeks (Fig. 2). The effectiveness of the hydrogels at inhibiting bacterial growth was compared in each instance to an antibiotic free hydrogel which had been stored under the same conditions. Vancomycin was used as a secondary control, as it is ineffective against *E. coli* ATCC 25922, and showed comparable results with the antibiotic free control throughout. When stored at room temperature, the majority of the antibiotic containing hydrogels, with the exception of colistin and piperacillin, were only effective up to 1 week of storage and after this point no significant difference could be observed from the antibiotic free controls.

### Antibiotic susceptibility testing

**Fig. 3:**
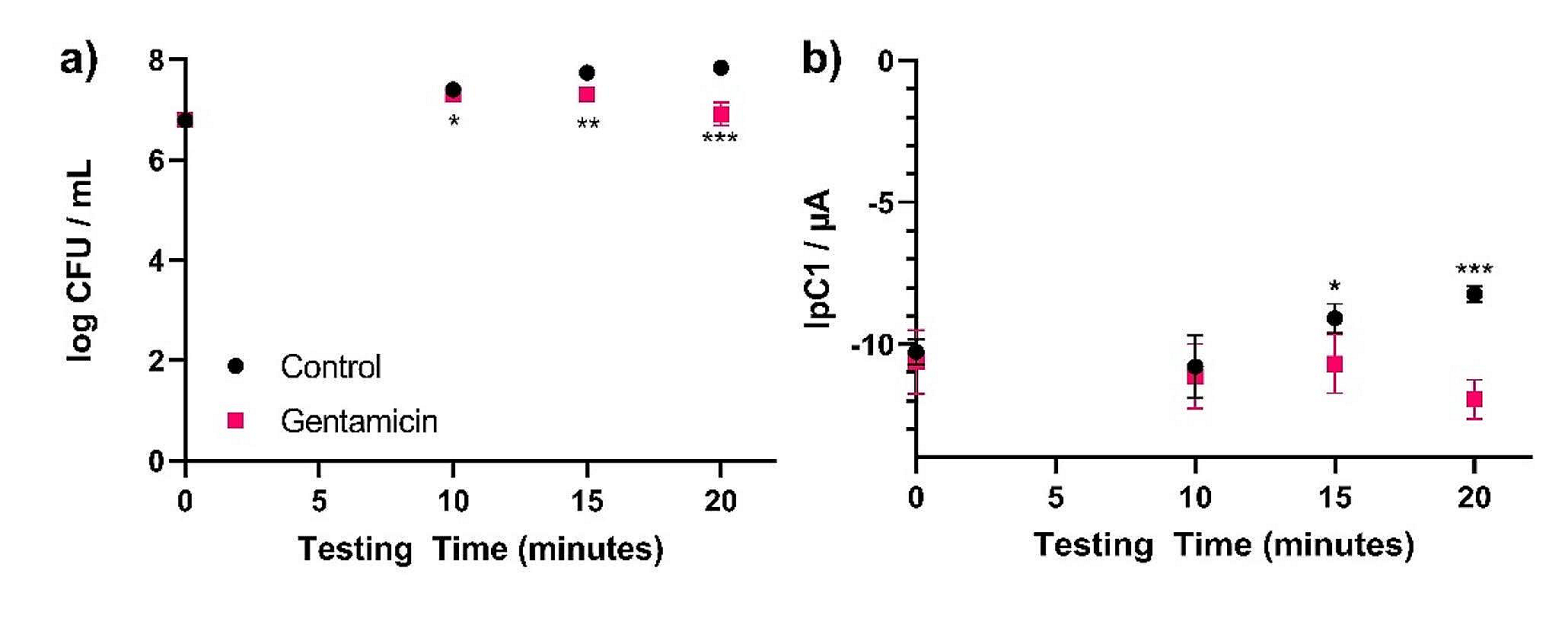
Antibiotic susceptibility testing results in the LOC device using gentamicin and antibiotic free hydrogels, comparing **(A)** CFU/mL counts and **(B)** resazurin reduction peak heights, for up to 20 min incubation (*n* = 4). Error bars represent standard deviation, significance is indicated as follows: * = p-value < 0.05, ** = p-value < 0.005, *** = p-value < 0.0005

Following evaluation of the antibiotic hydrogels, experiments were carried out to ensure that this mode of antibiotic delivery was compatible with the use of the SPEs in the LOC device. *E. coli* ATCC 25922 growth in artificial urine using gentamicin as an example antibiotic was compared to growth in antibiotic free controls and analysed using both traditional CFU counts as well as DPV (Fig. 3). Antibiotic susceptibility was observed from 10 min incubation with CFU counts and from 15 min incubation with DPV. For all subsequent LOC analysis, DPV scans were conducted after 15 min.

### LOC devices

In order to ensure that samples added to the LOC devices were evenly distributed across all 8 chambers, devices were filled and then solutions from each chamber were collected and CFU/mL determined across two different devices. No significant difference in CFU/mL count were obtained either between chambers on the same LOC device (F = 0.8742, DF = 7, *P* = 0.5289) or across different LOC devices (F = 1.998, DF = 1, *P* = 0.1599).

**Fig. 4:**
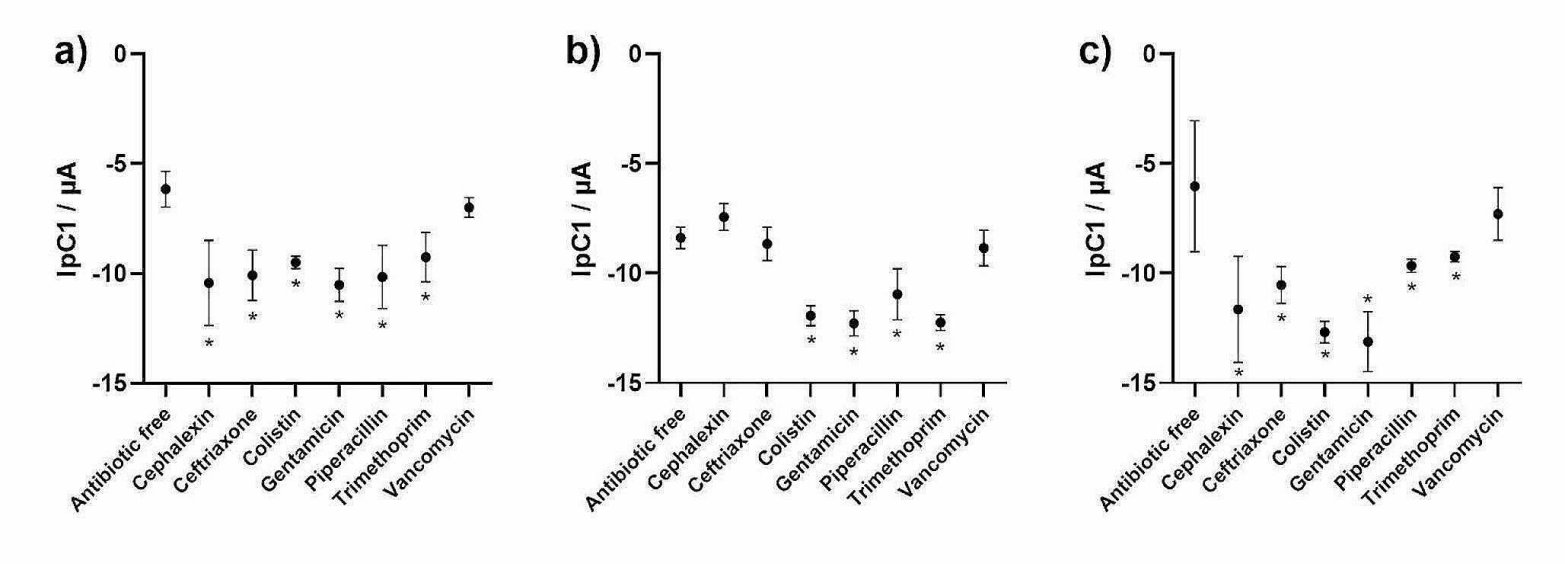
Resazurin IpC’s derived from the antibiotic-free control versus antibiotic hydrogels after 15 min of incubation for **(A)** *E. coli* ATCC 25922, **(B)** *E. coli* NCTC 13351, **(C)** *K. pneumoniae* ATCC 700603. DPV was conducted using SPEs (*n* = 4). Error bars represent standard deviation, significance is indicated as follows: * = p-value < 0.05

The combined LOC devices, with antibiotic hydrogels and SPEs, were then evaluated against three different bacterial species with different antibiotic susceptibility profiles spiked into artificial urine. The results show that antibiotic susceptibility could be determined within 15 min (following a 70 min pre-incubation) on the LOC devices by measuring the IpC values. As expected, *E. coli* ATCC 25922 (Fig. 4a) and *K. pneumoniae* ATCC 700603 (Fig. 4b) were only resistant to vancomycin, whereas *E. coli* NCTC 13351 (Fig. 4c) showed resistance to cephalexin, ceftriaxone and vancomycin. The limits of detection were evaluated, and antibiotic susceptibility could be determined from starting concentrations of as few as 1 × 10^5^ CFU/mL.

**Fig. 5:**
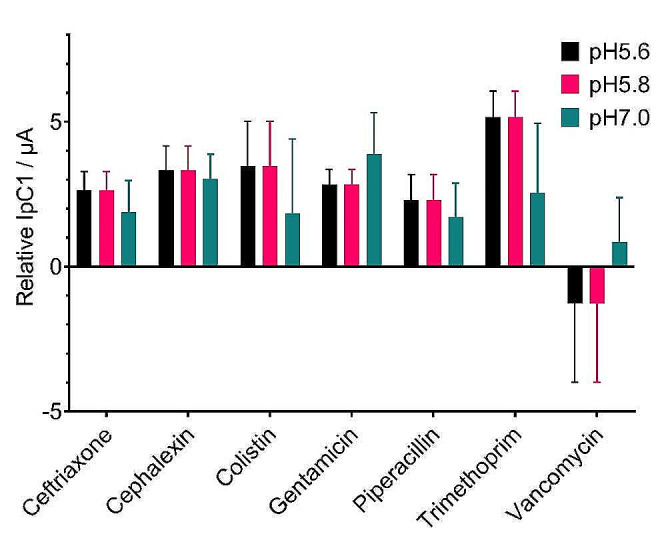
Resazurin IpC’s plotted relative to the antibiotic-free control (black line at 0) for each antibiotic tested. The example shown is for *K. pneumoniae* ATCC 700603 spiked in donated urine samples at pH 5.6, pH 5.8 and pH 7.0. DPV was conducted using R-SPEs (*n* = 4). Error bars represent standard deviation

These experiments were then repeated using donated urine samples to better mimic those which would be found in real patient specimens. Urine samples are heterogeneous and can vary depending on the hydration status, diet, and the taking of medications resulting in variation in elemental traces, metabolites, salt concentrations and pH (Merchant et al. 2017). The donated urine samples tested here ranged from pH 5.6 to 7.0 and were spiked individually with each of the three bacterial strains tested previously. The results showed that, while there are differences observed in the actual IpC values obtained across different pH values, when examined relative to the antibiotic-free control, the same profile of results regarding susceptibility to each antibiotic was consistent (Fig. 5).

### Discussion

The work presented here shows the development of a new method of conducting rapid AST using a LOC based electrochemical method. Resazurin is commonly used as redox indicator to detect metabolically active bacteria, but in this instance has been incorporated into SPEs which enables direct integration within the LOC device in a reproducible, sensitive and cost-effective manner. Antibiotics could be stored within the LOC devices in PVA hydrogels for up to four weeks in the freezer, resulting in release of the antibiotics into the appropriate chambers on the LOC device at the point where the urine sample is added. This aligns with a previous study which showed that minocycline and gentamicin containing agarose hydrogels were stable for 7 days at room temperature (Grolman et al. 2019). While there is widespread interest in the use of antibiotic hydrogels, there is limited literature describing the effects of long-term storage at different temperatures on antibiotic activity specifically within hydrogels. Here we found that cold storage was more effective and produced effective hydrogels after 4 weeks for both fridge and freezer conditions. The one exception was the storage of trimethoprim hydrogels stored in the fridge which were unable to produce an inhibitory effect. Due to this, and the desire to create an LOC in which the all the hydrogels can be housed prior to use, freezer storage was recommended.

The antibiotic susceptibility profiles of three different bacterial strains were established in both artificial and real urine matrices, within 85 min (15 min testing time plus 70 min pre-incubation). The LOC system was able to determine antibiotic susceptibility with a limit of detection of 1 × 10^5^ CFU/mL which is in line with clinically relevant concentrations found in human urine samples. The sensitivity reported here using SPEs is comparable to another microfluidic electrochemical device that used gold electrodes and demonstrated a limit of detection of 100 CFU/µL (Besant et al. 2015). The clinical CFU/mL count used in the diagnosis of a UTI can vary from ≥ 10^4^ − 10^6^ CFU/mL as the cut-off concentration is subjective and dependent on age, symptoms and urine collection method (Grabe et al. 2015; Coulthard et al. 2010; Fünfstück 1998; Schmiemann et al. 2010), but the limits of detection presented here using this methodology fall within this clinically relevant range and there is potential for further optimisation. When testing real urine samples, previous research has shown that urine samples contain components which have previously been shown to potentially affect electrochemical analysis, for example albumin and formate can become absorbed at the surface of the electrode, whereas creatinine and citrate are electrochemically active (Diouf et al. 2017). This natural heterogeneity of the urine samples may account for the differences in the magnitude of the signals observed, but this does not have a negative effect on the ability of the device to determine which antibiotics would be suitable for the patient.

The proposed LOC device offers advantages over current culture-based antibiotic susceptibility testing in terms of the speed of analysis whilst still maintaining clinically relevant levels of detection. The incorporation of SPEs compared to other electrochemical testing methods provides a more cost-effective and easily manufacturable solution, but there can be limitations associated with the reproducibility of printing the active surface area (Fanjul-Bolado et al. 2007). The work presented here demonstrates proof-of-concept using seven commonly prescribed antibiotics evaluated against 3 different bacterial strains which are most prevalent in uncomplicated UTIs. Future work would involve expanding this out to other antibiotics as prescribing practices change and testing a wider range of pathogens which are responsible for UTIs to ensure the LOC device is widely applicable. Rapid AST at point-of-care is made possible with the proposed microfluidic electrochemical method, which is essential for the timely and efficient prescription of appropriate antibiotics. This in turn can extend the longevity of antibiotics that are currently used in clinical settings, and any antibiotics introduced in the future.

## Electronic supplementary material

Below is the link to the electronic supplementary material.

Supplementary Material 1

## Data Availability

The raw data supporting the conclusions of the article will be made available by the authors, without undue reservation.
