# Supplementary material for: Multiplex antibiotic susceptibility testing of urinary tract infections using an electrochemical lab-on-a-chip
Source: Biomed Microdevices. 2024 Aug 9;26(3):35. doi: 10.1007/s10544-024-00719-w (PMC11315706; doi:10.1007/s10544-024-00719-w)
Supplement: Supplementary file 1 — Supplementary Material 1 [file 10544_2024_719_MOESM1_ESM.docx]

Title: Multiplex antibiotic susceptibility testing of urinary tract infections using an electrochemical lab-on-a-chip

Benjamin Crane^1^, Alex Iles (0000-0003-0837-6160)^2†^, Craig E. Banks (0000-0002-0756-9764)^1^, Mamun Rashid (0000-0002-9769-2431)^1^, Patricia E. Linton (0000-0002-1106-4988)^1^, Kirsty J. Shaw (0000-0001-9241-4195)^1^*


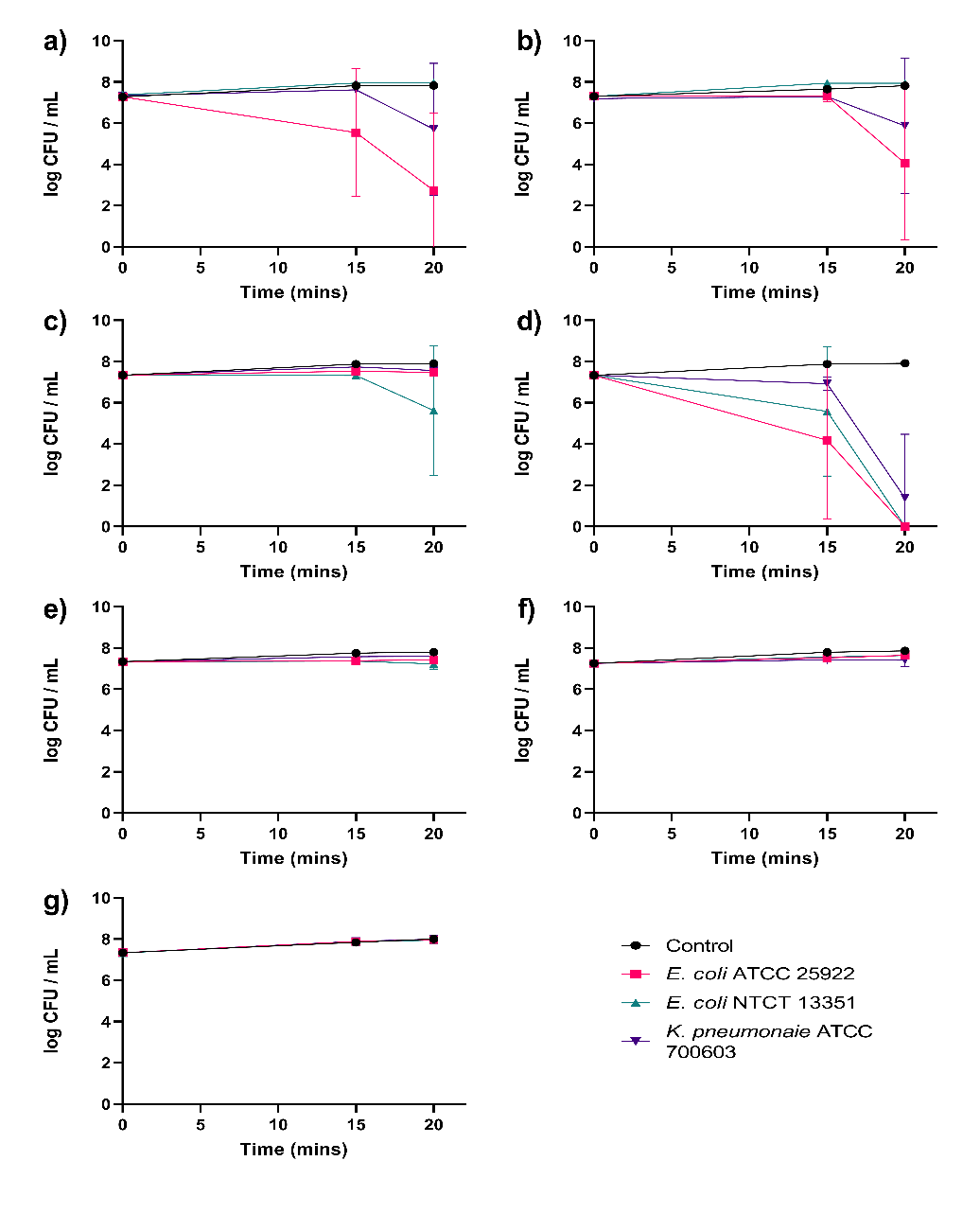


***Fig. SI_1*** *Bacterial growth of E. coli ATCC 25922, K. pneumoniae ATCC 700603 and E. coli NCTC 13351 in response to different antibiotic loaded hydrogels:* ***(A)*** *ceftriaxone;* ***(B)*** *cephalexin;* ***(C)*** *colistin;* ***(D)*** *gentamicin;* ***(E)*** *piperacillin;* ***(F)*** *trimethoprim; and* ***(G)*** *vancomycin. Antibiotic-free control hydrogels are included as part of each experiment (n=5, error bars represent standard deviation)*
